# Epidemiology of synchronous brain metastases

**DOI:** 10.1093/noajnl/vdaa041

**Published:** 2020-04-24

**Authors:** Raj Singh, Kelsey C Stoltzfus, Hanbo Chen, Alexander V Louie, Eric J Lehrer, Samantha R Horn, Joshua D Palmer, Daniel M Trifiletti, Paul D Brown, Nicholas G Zaorsky

**Affiliations:** 1 Department of Radiation Oncology, Virginia Commonwealth University Health System, Richmond, Virginia, USA; 2 Department of Radiation Oncology, Penn State Cancer Institute, Hershey, Pennsylvania, USA; 3 Department of Public Health Sciences, Penn State College of Medicine, Hershey, Pennsylvania, USA; 4 Department of Radiation Oncology, London Health Sciences Centre, London, Ontario, Canada; 5 Department of Radiation Oncology, Odette Cancer Centre—Sunnybrook Health Sciences Centre, Toronto, Ontario, Canada; 6 Department of Radiation Oncology, Icahn School of Medicine at Mount Sinai, New York, New York, USA; 7 Department of Radiation Oncology, The James Cancer Hospital at the Ohio State University Wexner Medical Center, Columbus, Ohio, USA; 8 Department of Radiation Oncology, Mayo Clinic, Jacksonville, Florida, USA; 9 Department of Radiation Oncology, Mayo Clinic, Rochester, Minnesota, USA

**Keywords:** brain metastases, cancer, epidemiology, incidence, prognosis

## Abstract

**Background:**

The objectives of this study were to characterize (1) epidemiology of brain metastases at the time of primary cancer diagnosis, (2) incidence and trends of synchronous brain metastases from 2010 to 2015, and (3) overall survival (OS) of patients with synchronous brain metastases.

**Methods:**

A total of 42 047 patients with synchronous brain metastases from 2010 to 2015 were identified from the Surveillance, Epidemiology, and End Results database. Descriptive analysis was utilized to analyze demographics and incidence. The Kaplan–Meier method and a Cox proportional hazards model were utilized to evaluate potential prognostic factors for OS.

**Results:**

The majority of patients were diagnosed from age older than 50 (91.9%). Common primary sites included lung (80%), melanoma (3.8%), breast (3.7%), and kidney/renal pelvis (3.0%). Among pediatric patients, common primaries included kidney/renal pelvis and melanomas. The incidence was roughly 7.3 persons/100 000. Synchronous brain metastases were associated with significantly poorer OS compared to extracranial metastases alone (hazard ratio [HR] =1.56; 95% CI: 1.54–1.58; *P* < .001). Among patients with brain metastases, male gender (HR = 1.60 vs 1.52), age older than 65 years (HR = 1.60 vs 1.46), synchronous liver, bone, or lung metastases (HR = 1.61 vs 1.49), and earlier year of diagnosis (HR = 0.98 for each year following 2010) were associated with significantly poorer OS.

**Conclusions:**

The vast majority of brain metastases are from lung primaries. Synchronous brain metastases are associated with poorer OS compared to extracranial metastases alone.

**Key Points**

- The incidence of synchronous brain metastases was 7.3/100 000; roughly 80% were from lung primaries.
- Brain metastases are associated with poorer survival among those with extracranial metastatic disease.
- Older age, male gender, and extracranial metastases are associated with poorer survival among patients with brain metastases.

**Importance of the Study**

We sought to analyze modern incidences and demographics of patients with synchronous brain metastases, examine the impact of brain metastases on survival for patients with metastatic disease, and evaluate potential factors associated with survival among patients with brain metastases in the United States utilizing the SEER database. From 2010 to 2015, we noted that there was no significant change in the incidence of brain metastases at primary cancer diagnosis, with a predominance of brain metastases (roughly 80%) from lung primaries and cancers of the kidney/renal pelvis and cutaneous melanomas being common primary sites among pediatric patients. Patients with metastatic disease with brain metastases were noted to have poorer survival as compared to patients with extracranial metastases alone. Also, patients with brain metastases who were older than 65 years, of male gender, diagnosed earlier in the time period studied, or also had liver, bone, or lung metastases had significantly poorer survival.

Brain metastases account for the majority of intracranial tumors and constitute the disease course of anywhere from 15% to 20% of adults and 5–10% of children with malignancies. Estimates of new diagnoses of brain metastases each year in the United States range anywhere from roughly 30 000 to 40 000 patients.^1–3^ The most common primary sites in adults have previously been shown to be cancers of the lung, kidney, breast, and colon, in addition to melanomas; in the pediatric population common primary sites are generally thought to be sarcomas, germ cell tumors, and neuroblastomas.^1–5^ The morbidity and mortality associated with brain metastases are generally quite poor, with associated neurologic deficits either from mass effect secondary to the tumors themselves or therapy-related toxicities, and median overall survival (OS) ranging from 3 to 15 months.^6–9^

Notably, the incidence of brain metastases has reportedly risen over the past few decades for a number of primary cancer sites, particularly among patients with breast cancers,^10^ colorectal cancers,^11^ and non-small-cell lung cancer (NSCLC).^12^ However, these observations are limited to single-institution reports, smaller multi-institutional series, or patients enrolled on clinical trials.^6,13^ Previous studies have examined the epidemiology of brain metastases in Detroit and The Netherlands.^14,15^ There are currently no data on the epidemiology of brain metastases among all cancer patients in the United States.

We sought to address this gap in the literature regarding patients with synchronous brain metastases (defined as patients diagnosed with brain metastases at primary cancer diagnosis) with 3 main aims. First, we aimed to examine the demographics of patients with synchronous brain metastases, with an emphasis on patient age, gender, race, and primary cancer site. Second, we analyzed recent trends in the incidence of synchronous brain metastases in the United States among the general population, as well as by primary cancer site and age group at diagnosis. Finally, we sought to compare the OS of patients with synchronous brain metastases to patients with extracranial metastases alone at primary cancer diagnosis as well as examine for any factors associated with OS among patients with brain metastases.

## Materials and Methods

### Data Acquisition

Patients with brain metastases at the time of primary cancer diagnosis, diagnosed between 2010 and 2015 (2010 is when information on brain metastases began to be queried), were captured from the National Cancer Institute’s Surveillance, Epidemiology, and End Results (SEER) program. For the purposes of the analysis to compare demographics, patients without brain metastases were also queried from the SEER database. SEER is a network of population-based incidence tumor registries that covered 27.8% of the US population consisting of geographically distinct regions of the United States at the time of data analysis.^16^ Prior reports have noted both strengths and limitations of the SEER database, with strengths including a large cohort of patients available for analysis allowing for generalizability to the US population as well as quality control programs to minimize miscoding of patient information.^16–18^

SEER*Stat 8.3.5 was used for analysis. Patients diagnosed by autopsy or death certificate alone were excluded from the analysis. The SEER 18 registry (2000–2015) was used for the current analysis, including both the case listing and rate sessions.^16^ All incidence rates were age-adjusted to the 2000 US standard population and are reported per 100 000 persons. Additional analyses were conducted using Microsoft Excel 15.0.5 (Microsoft) and R Studio (R Studio Inc.).

### Statistical Analyses

For all patients with brain metastases, demographics were categorized by age at diagnosis, race, sex, and primary site. Furthermore, patients with and without de novo brain metastases were compared by age, sex, year of diagnosis, presence of non-brain metastases, race, T stage, N stage, median follow-up duration, and primary cancer site. To assess differences in these categories between patients with and without brain metastases, *t*-tests, chi-squared tests, or log-rank tests for continuous, categorical, and time to event variables, respectively, were used where appropriate. Additionally, trends in incidence rates over the study time period (2010–2015) were assessed by the primary cancer site. A weighted least-squares method was used to calculate annual percentage changes (APCs) with SEER*Stat. While all cancer sites were included in the analysis, only primary cancers with an incidence rate greater than 0.05 per 100 000 persons from 2010 to 2015 are presented to simplify interpretation. A Bonferroni correction was applied to all analyses other than those stratified by primary sites testing 9 hypotheses, which resulted in statistical significance being set at *P* < .006.

For the survival analysis, the population was defined as all cases with de novo metastatic cancer (M1 by the American Joint Committee on Cancer 7th edition definition). The main exposure was the presence of synchronous brain metastasis as a dichotomous variable and the main outcome was OS, which was estimated by the Kaplan–Meier method. Median follow-up was estimated using the reverse Kaplan–Meier method.^19^ Kaplan–Meier curves were created to compare OS between metastatic patients with synchronous brain metastases (with or without other metastases at primary diagnosis) and patients with extracranial metastases alone at primary cancer diagnosis (lung, liver, and/or bone with no brain metastasis). Univariate Cox proportional hazards regressions were used to compare the hazards of death for patients with synchronous brain metastases versus those with extracranial metastases alone. Potential confounders of age (continuous), sex (dichotomous), race (categorical), year of diagnosis (categorical), T- and N-stage (categorical), and the presence of other metastases (dichotomous) were adjusted for with a multivariable Cox regression model. Effect modification for OS among patients with brain metastases by age, sex, race, year of diagnosis, and the presence of other metastases (bone, liver, or lung) was investigated by the addition of interaction terms to multivariable Cox regression models. For lung and breast cancer patients, the effect of different cancer subtypes (either NSCLC or small-cell lung cancer [SCLC] as well as hormone receptor [HR] positive or negative and HER2-neu positive or negative) was also investigated with interaction terms. The proportional hazards assumption was assessed using Schoenfeld residual tests and Log(HR) versus time plots for the overall and site-specific multivariable regression models. Results stratified by primary site of origin were subject to Holm’s correction for multiple testing, with a threshold for statistical significance of .05 post-adjustment.^20,21^

## Results

### Patient Demographics Stratified by Age and Gender

A flowsheet of how patients were selected for inclusion for analysis can be seen in Supplementary Figure 1. We identified a total of 42 047 patients with synchronous brain metastases from 2010 to 2015 with 41 105 patients available for survival analysis after the exclusion of those without information on other sites of metastatic disease or OS. A total of 2 056 647 patients without brain metastases were also identified for means of comparing baseline demographics. A summary of patient characteristics with synchronous brain metastases by age, race, sex, and cancer site broken down by year and with overall statistics over the time period studied can be found in Supplementary Table 1. With regard to age, the most common age group in which a diagnosis of brain metastasis was made was from 60 to 69 years old (33%), followed by 70–79 years old (24.5%), 50–59 years old (23.3%), older than 80 years (11.2%), and younger than 50 years (8.11%). Males comprised 52.5% of the cohort examined, with whites, blacks, and other races comprising 80.1%, 12%, and 7.8% of patients, respectively. Additionally, we examined differences in demographics between patients with synchronous brain metastases as compared to all other patients without brain metastases, which is provided in Table 1. Patients with synchronous brain metastases were more likely to be older, male, non-white, and have more advanced T and N staging (all *P* < .001).

**Table 1.** Summary of Baseline Characteristics of Patients With Brain Metastases as Compared to Patients Without Brain Metastases

	Brain Metastasis (*N* = 42 047)	No Brain Metastasis (*N* = 2 056 647)	*P*
Age, mean (standard deviation)	64.99 (12.02)	64.22 (14.50)	<.001^a^
Sex			
Female	19 989 (47.5%)	1 040 294 (51.6%)	<.001^b^
Male	22 058 (52.5%)	1 016 353 (49.4%)	
Year of diagnosis			
2010	6699 (15.9%)	334 313 (16.3%)	
2011	6734 (16.0%)	339 201 (16.5%)	
2012	6961 (16.6%)	339 801 (16.5%)	<.001^b^
2013	7114 (16.9%)	342 544 (16.7%)	
2014	7306 (17.4%)	347 392 (16.9%)	
2015	7233 (17.2%)	353 395 (17.2%)	
Race			
American Indian/ Alaska Native	249 (0.6%)	12 127 (0.6%)	
Asian or Pacific Islander	2998 (7.1%)	137 248 (6.7%)	<.001^b^
Black	5034 (12.0%)	221 846 (10.8%)	
White	33 692 (80.1%)	1 658 355 (80.6%)	
Unknown	74 (0.2%)	27 071 (1.3%)	
T-stage			
T0	1201 (2.9%)	6567 (0.3%)	
T1	4446 (10.6%)	811 899 (39.5%)	
T2	9144 (21.7%)	478 510 (23.3%)	<.001^b^
T3	8348 (19.9%)	337 164 (16.4%)	
T4	10 894 (25.9%)	160 678 (7.8%)	
TX	6999 (16.6%)	137 323 (6.7%)	
Missing	1015 (2.4%)	62 048 (3.0%)	
Other T	0 (0%)	62 458 (3.0%)	
N-stage			
N0	10 155 (24.2%)	1 433 217 (69.7%)	
N+	26 937 (64.1%)	491 890 (23.9%)	<.001^b^
NX	3927 (9.3%)	69 316 (3.4%)	
Missing	1028 (2.4%)	1370 (0.1%)	
M-stage			<.001^b^
M1	1033 (97.6%)	293 557 (14.3%)	
M0	0 (0%)	1 701 063 (82.7%)	
Missing	1014 (2.4%)	62 027 (3.0%)	

^a^Two-sample *t*-tests.

^b^Chi-squared tests.

**Figure 1.**
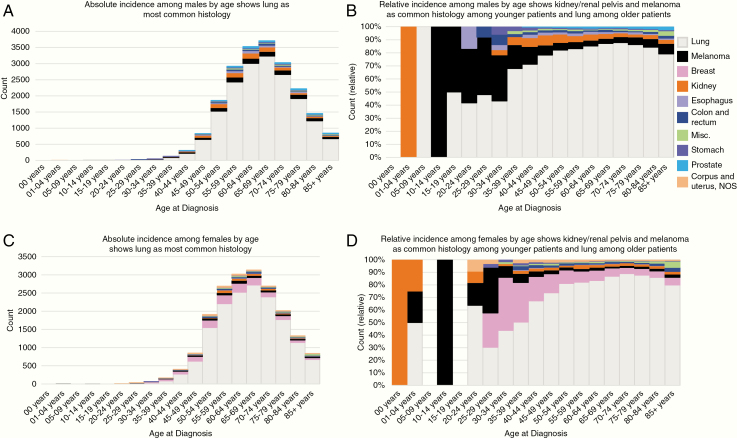
Incidence of brain metastases among males and females, by age, from SEER 2010 to 2015. (A) Absolute incidence among males by age shows lung as the most common histology. (B) Relative incidence among males by age shows kidney/renal pelvis and melanoma as common histology among younger patients and lung among older patients. (C) Absolute incidence among females by age shows lung as the most common histology. (D) Relative incidence among females by age shows kidney/renal pelvis and melanoma as common histology among younger patients and lung among older patients.

We also examined the incidence of synchronous brain metastases by age at diagnosis for males and females separately, shown in Figure 1A and B and Figure 1C and D, respectively. Among male patients 1–4 years of age and female patients less than 1 year of age, tumors of the kidney and renal pelvis were the only primary site associated with brain metastases. Melanomas and lung cancers were the most common primary sites from ages 10–14 and 15–29 years old, respectively, in the male cohort. Similar to male pediatric patients, melanomas were the most common primary sites among female patients aged 10–14. Cancers of the lung comprised the majority of primary sites for both male and female patients aged 35 years and older with brain metastases as the time of primary cancer diagnosis.

### Trends in Incidences of Synchronous Brain Metastases Stratified by Disease Site and Age

**Figure 2.**
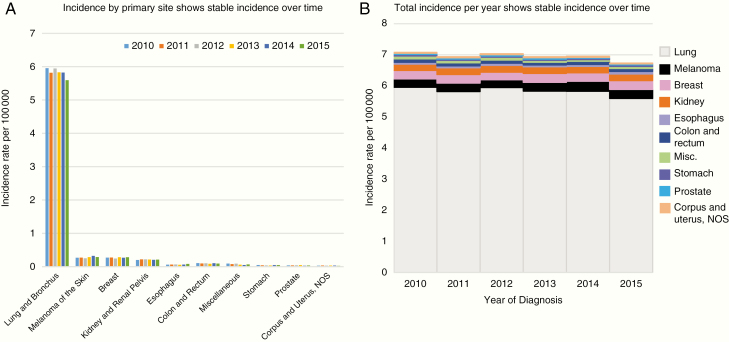
Incidence of brain metastases by primary site. (A) Incidence by the primary site shows a stable incidence over time. (B) The total incidence per year shows a stable incidence over time.

Trends in brain metastases by the primary site over time can be found in Supplementary Table 2 and Figure 2. The most common primary site was consistently lung (with an incidence ranging from 5.596 to 5.954 per 100 000, or roughly 80% of all brain metastases) from 2010 to 2015, with melanoma (0.252–0.324 per 100 000; 3.8%) and breast (0.244–0.284 per 100 000; 3.7%) being the second and third most common sites, followed closely by cancers of the kidney and renal pelvis (0.206–0.22 per 100 000; 3.0%). Upon examination of lung and bronchus by subtype, we noted an incidence of roughly 4.0–4.3 persons per 100 000 among NSCLC patients and 0.92–0.99 persons per 100 000 among SCLC patients (Supplementary Figure 2). The incidence of synchronous brain metastases across all primary sites ranged from roughly 7.1 to 7.4 persons per 100 000 and was relatively constant throughout the study time period among all sites. A similar trend was observed when looking at trends over time by the primary site. A nonsignificant decline in brain metastasis diagnosis from all primary sites during the time period was noted (−0.59% APC; *P* = .09) with the majority of this decline accounted for by brain metastases from lung cancers (−0.932% APC; *P* = .07) with no significant changes in incidence noted of any primary site. In Supplementary Table 3, trends in age-adjusted incidences of synchronous brain metastases by age at diagnosis are given, which noted significant increases in incidences of brain metastases among those aged 35–39 (*P* = .004) and declines among those aged 45–49 (*P* = .002) and 50–54 (*P* = .003).

### Survival of Patients With Metastatic Disease Based on Disease Site and Presence of Synchronous Brain Metastases or Extracranial Metastases Alone

We first examined OS outcomes of patients with synchronous brain metastases based on the primary site. Kaplan–Meier estimate survival curves for the 6 most common primary cancer sites based on whether patients had brain or extracranial metastases alone can be found in Supplementary Figure 3, with additional Kaplan–Meier curves for the next 6 most common sites in Supplementary Figure 4. Of the 6 most common primary sites, patients with breast cancer and brain metastasis had the highest 1-year OS rate of 41.4% and a median OS of 8 months. One-year OS rates and median OS for other common sites were as follows: melanoma (24.7%; 4.9 months), kidney and renal pelvis (23.7%; 3.9 months), and lung (20.3%; 3.9 months). Among patients with brain metastases, other common sites associated with higher 1-year OS rates included prostate (48.7%) and testes (52.7%), with poorer outcomes in patients with pancreas (7.5%), bladder (10.9%), and stomach (15.0%) cancers, compared to the respective cancer population with extracranial metastases. We also examined OS based on SCLC and NSCLC histologies, which can be seen in Supplementary Figure 5 as well as for breast cancer by receptor status, which is presented in Supplementary Figure 6. Of note, a significant difference in OS was noted for patients with NSCLC with or without brain metastases (*P* < .0001) but not among patients with SCLC (*P* = .68). Poorer OS was noted among all patients with breast cancer with synchronous brain metastases of any hormonal receptor subtype (*P* < .0001).

We also analyzed for potential differences between patients with metastatic disease either with or without synchronous brain metastases. Patients with synchronous brain metastases were found to be significantly younger, have lower rates of lung, bone, or liver metastases at diagnosis, were more likely to be white, and had more advanced T and N stage with significant differences in primary site (*P* < .001) (Supplementary Table 4). With regard to OS, as given in Table 2, on univariate analysis the hazard ratio (HR) for OS for patients with synchronous brain metastases was significantly higher (HR = 1.43 [95% CI: 1.41–1.44]; *P* < .001). Following multivariable analysis controlling for potential confounders across all primary sites, patients with synchronous brain metastases had significantly poorer OS as compared to those with extracranial metastases alone (HR = 1.56 [95% CI: 1.54–1.58]; *P* < .001).

**Table 2.** Results of Univariate and Multivariate Cox Regression Comparing the Hazards of Death of Patients With Extracranial Metastases Alone or With Brain Metastases

	HR	95% CI	*P*
Univariate analysis			
Brain metastasis vs extracranial metastases alone	1.43	[1.41–1.44]	<.001
Multivariable analysis			
Brain metastasis vs extracranial metastases alone	1.56	[1.54–1.58]	<.001
Male vs female sex	1.13	[1.12–1.14]	<.001
Year of diagnosis (per each year increase)	0.98	[0.98–0.98]	<.001
Age (per each year increase)	1.02	[1.02–1.02]	<.001
T-stage			
T0 vs T1	1.1	[1.06–1.14]	<.001
T2 vs T1	1.18	[1.16–1.20]	<.001
T3 vs T1	1.12	[1.10–1.13]	<.001
T4 vs T1	1.31	[1.29–1.33]	<.001
TX vs T1	1.51	[1.48–1.53]	<.001
N-stage			
N+ vs N0	1.15	[1.14–1.17]	<.001
NX vs N0	1.18	[1.17–1.20]	<.001
Race			
American Indian/Alaska Native vs white	1.05	[1.00–1.11]	.036
Asian/Pacific Islander vs white	0.86	[0.85–0.87]	<.001
Black vs white	1.08	[1.06–1.09]	<.001
Unknown vs white	0.51	[0.46–0.57]	<.001
Coexisting lung/bone/ liver metastasis vs no lung/bone/liver metastasis	1.15	[1.14–1.16]	<.001

HR, hazard ratio; CI, confidence interval.

**Figure 3.**
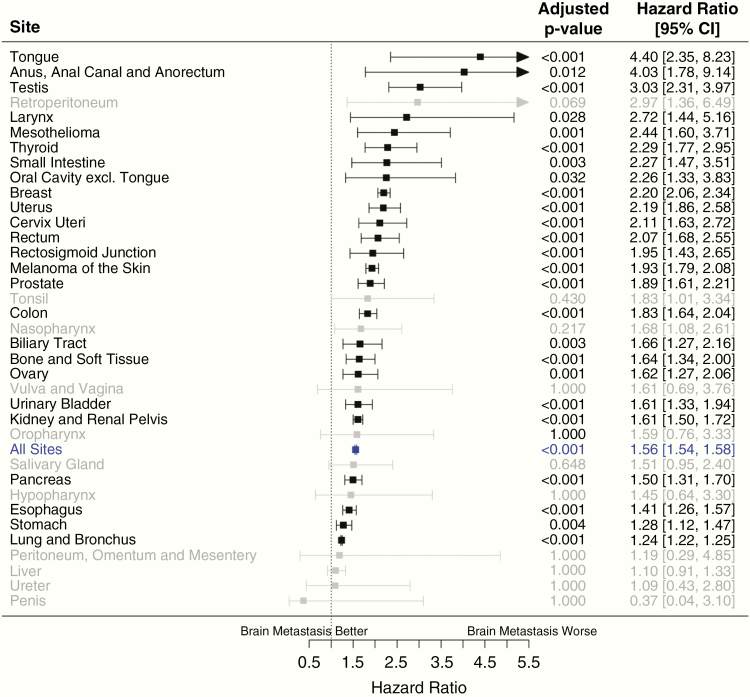
Forest plots showing poorer overall survival (OS) among patients with brain metastases compared to extracranial metastases alone by the primary site.

Figure 3 demonstrates differences in OS by the primary site for patients with metastatic disease either with or without brain metastases (all *P* < .001). The most marked differences in OS between patients with either synchronous brain metastases or extracranial metastases alone were noted for cancers of the tongue, anus/anal canal/anorectum, and testis. Of the top 12 most common sites with brain metastases, patients with breast cancer and brain metastases had the poorest OS as compared to those with extracranial metastases alone (HR = 2.20 [95% CI: 2.06–2.34]), followed closely by uterine cancers (HR = 2.19 [95% CI: 1.86–2.58]), rectal cancers (HR = 2.07 [95% CI: 1.68–2.55]), and melanomas (HR = 1.93 [95% CI: 1.79–2.08]). For breast cancer patients, the detrimental effect of synchronous brain metastases on OS was highest in HER2+ and HR− patients (HR = 2.47 [95% CI: 2.02–3.01]), followed by HER2− and HR+ patients (HR = 2.23 [95% CI: 2.01–2.47]), triple-negative patients (HR = 2.00 [95% CI: 1.75–2.29]), and HER2+ and HR+ patients (HR = 1.93 [95% CI: 1.62–2.31]; *P* < .001). Differences in OS for lung primaries were not as marked but still quite significant (HR = 1.24 [95% CI: 1.22–1.25]) and were maintained on analysis of both SCLC (HR = 1.15 [95% CI: 1.11–1.19]) and NSCLC (HR = 1.26 [95% CI: 1.24–1.28]) histologies. Median OS examined by primary cancer site as well as whether patients had extracranial metastases alone or brain metastases at diagnosis can be found in Table 3.

**Table 3.** Number of Patients With or Without Synchronous Brain Metastases at Diagnosis by Primary Cancer Site and Associated Unadjusted Median Survival Time in Months

Cancer Site	All Cancer Patients (M0 and M1) Diagnosed 2010–2015		M1 Patients With Brain Metastasis at Diagnosis		M1 Patients Without Brain Metastasis at Diagnosis	
	*N*	Median Survival, Months (95% CI)	*N* (%)	Median Survival, Months (95% CI)	*N* (%)	Median Survival, Months (95% CI)
Lung and bronchus	308 561	10 (10-10)	33 668 (10.9%)	4 (4-4)	104 664 (33.9%)	4 (4-4)
Small cell	36 281	7 (7-7)	5558 (15.3%)	5 (4–5)	17 790 (49.0%)	5 (5-5)
Non-small cell	272 280	11 (11-11)	28 110 (10.3%)	4 (4-4)	86 874 (31.9%)	4 (4-4)
Melanoma	126 397	NR (NR-NR)	1595 (1.3%)	5 (4–5)	2918 (2.3%)	12 (11–12)
Breast	379 261	NR (NR-NR)	1562 (0.4%)	9 (7–10)	19 208 (5.1%)	28 (28–29)
HR+/HER2-	253 783	NR (NR-NR)	580 (0.2%)	12 (10–15)	9910 (3.9%)	34 (33–35)
HR+/HER2+	36 458	NR (NR-NR)	225 (0.6%)	20 (15–29)	2543 (7.0%)	43 (41–46)
HR−/HER2+	15 727	NR (NR-NR)	176 (1.1%)	10 (8–14)	1328 (8.4%)	34 (30–41)
HR−/HER2−	39 147	NR (NR-NR)	287 (0.7%)	5 (4–6)	2160 (5.5%)	13 (12–13)
Unknown	34 146	NR (NR-NR)	294 (0.9%)	2 (2–4)	3267 (9.6%)	14 (13–15)
Kidney	89 382	NR (NR-NR)	1253 (1.4%)	4 (4–5)	11 685 (13.1%)	9 (9-9)
Colon and rectum	223 842	70 (69-NR)	573 (0.3%)	4 (3–5)	41 724 (18.6%)	13 (13-13)
Esophagus	24 033	11 (10–11)	393 (1.6%)	3 (3–4)	6536 (27.2%)	5 (5-5)
Stomach	40 565	14 (14–15)	253 (0.6%)	3 (2–4)	12 126 (29.9%)	5 (5-5)
Prostate	316 724	NR (NR-NR)	220 (0.1%)	11 (9–14)	17 136 (5.4%)	26 (25–27)
Corpus and uterus, NOS	80 623	NR (NR-NR)	186 (0.2%)	3 (3–4)	6073 (7.5%)	12 (12–13)

NR, not reached at the end of follow-up; HR, hormone receptor; HER2, human epidermal growth factor receptor 2.

When examining for effect modifications in OS for patients with synchronous brain metastases by other factors of interest, we noted poorer OS in males (HR = 1.60 [95% CI: 1.58–1.63]) compared to females (HR = 1.52 [95% CI: 1.49–1.54]; *P* for interaction < .001), patients aged 65 years or older (HR = 1.60 [95% CI: 1.58–1.63]; *P* < .001) compared to younger patients (HR = 1.46 [95% CI: 1.44–1.48]), and those with lung, bone, or liver metastases (HR = 1.61 [95% CI: 1.59–1.64]; *P* < .001) versus those without (HR = 1.49 [95% CI: 1.46–1.52]). Interestingly, patients diagnosed with synchronous brain metastases in 2010 had significantly poorer OS (HR = 1.62 [95% CI: 1.59–1.65]) versus those diagnosed in 2015 (HR = 1.49 [95% CI: 1.46–1.53]; *P* < .001) with an estimated improvement in OS over time (HR = 0.98 [95% CI: 0.98–0.99] for each year after 2010).

We also note that the effect of synchronous brain metastases on OS was not constant over time. The global Schoenfeld residual test for the multivariable Cox regression model for all sites was statistically significant (*P* < .001). A plot of the logarithm of the HR for this model as a function of time can be found in Supplementary Figure 7. The detrimental effect of synchronous brain metastases on OS reached a maximum approximately 3 months after diagnosis, followed by a rapid drop-off until 10 months after diagnosis then tapered off more slowly. The detrimental effect of brain metastases on OS remained statistically significant at the .05 level until the end of the follow-up period. On site-specific analysis, the Schoenfeld residual test was significant for only lung cancer (*P* < .001), breast cancer (*P* < .001), and melanoma (*P* = .042) after multiple testing adjustment. Log(HR) versus time plots for all available disease sites can be found in Supplementary Figure 8.

## Discussion

Brain metastases have a significant impact in the disease course of multiple cancer sites and are expected to have a rising incidence given improved systemic therapies and subsequently longer OS.^10–12^ As such, the importance of understanding the epidemiology and trends of brain metastases in the United States is paramount to guide future studies and clinical trials. Based on our findings, the majority of synchronous brain metastases across all age groups appear to be from lung primaries (roughly 80%) with a stable incidence of roughly 7 persons per 100 000 individuals at initial presentation. In the pediatric population, melanomas in patients from 10 to 14 years of age as well as those of the kidney and renal pelvis appear to comprise the majority of primaries. Notably, we also found that patients with synchronous brain metastases had significantly poorer OS as compared to patients with extracranial metastases alone (HR = 1.56 [95% CI: 1.54–1.58]; *P* < .001), with HRs for the most common sites ranging from an HR = 1.24 (95% CI: 1.22–1.25) for lung cancers to 2.20 (95% CI: 2.06–2.34) for breast cancers. Patients who were male, elderly, and had synchronous liver, bone, or lung metastases were noted to have a poorer OS.

Historical reports in both the United States and Scotland have previously noted incidence rates ranging from 8.3 to 14.3 brain metastasis persons per 100 000 individuals.^22–25^ Walker et al.^24^ noted a higher incidence of males with new brain metastases (9.7 males vs 7.1 females per 100 000 individuals) that was attributed to higher rates of lung primaries among male patients. However, Counsell et al.^25^ found a similar incidence rate among both sexes in their cohort. Similar to our findings, they also noted a gradual increase in the incidence rate of brain metastases by increasing age until the age of 74, when a sharp decline was noted, which was thought to be secondary to no formal workup for brain metastases.

Regarding primary cancer sites, a prior investigation by Barnholtz-Sloan et al.^14^ of the Metropolitan Detroit Cancer Surveillance System also found lung cancers to be the most common primary cancer site, though not constituting the majority of patients as in our study (19.9% of their cohort). Other common sites were melanoma (6.9%), breast (5.1%), and colorectal cancers (1.8%). Similar to these findings, a report by Schouten et al.^15^ from The Netherlands noted a 5-year cumulative incidence of new brain metastases to be highest among patients with lung cancers (16.3%), followed by renal cancers (9.8%), melanomas (7.4%), breast cancers (5%), and colorectal cancers (1.2%). Another report by Berghoff et al.^26^ of 2419 patients with brain metastases found that those with lung cancers comprised the largest proportion of solid tumor types with synchronous brain metastases (47%).

We noted a significantly higher rate of synchronous brain metastases associated with lung cancers (roughly 80%) similar to that of previous investigations utilizing the SEER database. Notably, previous analysis by Cagney et al.^27^ reported that patients with NSCLC or SCLC had the highest rates of brain metastases at initial diagnosis. Their analysis also found that of patients with metastatic disease at presentation, those with melanoma (28%), NSCLC-adenocarcinoma (26.8%), non-specified NSCLC or other lung cancers (25.6%), SCLC (23.5%), NSCLC-squamous cell carcinoma (15.9%), bronchioloalveolar carcinoma (15.5%), and renal cancers (10.8%) had the highest incidence of brain metastases. Another prior analysis of the SEER database by Kromer et al.^28^ examined patients with synchronous brain metastases from 2010 to 2013 and similarly noted the highest frequency among patients with cancers of the lung and bronchus (10.8%), and notably with SCLC noted to have the highest incidence among all histologies (15.1%).

Among the pediatric population, we noted that tumors of the kidney and renal pelvis was the only primary site for patients less than 1 year of age, with melanomas being the predominant site for older pediatric patients. Prior studies have noted brain metastasis incidence rates ranging from 1.5% to 2.5% among children with solid tumors, most commonly germ cell tumors and sarcomas (often Ewing’s sarcoma and osteosarcoma).^2,3^ Rates of brain metastases on prior reviews have been found to be as high as 13.5% (germ cell tumors), 6.5% (osteosarcomas), 3.3% (Ewing’s sarcomas), and 1.9% (rhabdomyosarcomas).^29^ The findings of our study may be different than prior reports as pediatric patients with sarcomas may develop brain metastases later on in their disease course rather than at initial diagnosis, which the SEER database would not have captured.

With respect to prognosis for patients with brain metastasis, our work noted that patients with brain metastases from breast cancers had a higher 1-year OS rate (41%) and a median OS of 8 months as compared to other common primary sites such as melanoma, kidney and renal pelvis, and lung (all with 1-year OS rates and median OS ranging from roughly 20% to 25% and 3.9 to 4.9 months, respectively). These findings are similar to those of Cagney et al.^27^ who noted that breast cancer patients had higher median OS (10 months) as compared to other primary sites, with other favorable primary sites being prostate (12 months) and bronchioalveolar carcinoma (10 months). Also, Berghoff et al.^26^ found in their brain metastasis cohort that patients with breast cancer had the longest median OS (8 months) as compared to patients with primaries of the lung or kidney (7 months), melanoma (5 months), or colon/rectum (4 months; *P* < .001).

Other prior studies have utilized multi-institutional cohorts or patients on prior prospective trials to develop a method to estimate patients’ prognosis following a brain metastasis diagnosis. One of the initial recursive partitioning analyses was reported by Gaspar et al.^13^ and examined 1200 patients with brain metastases from 3 Radiation Therapy Oncology Group prospective trials from 1979 to 2003 that defined 3 prognostic Classes (1–3) based on Karnofsky performance status score, age, and the presence or absence of extracranial metastases for patients with brain metastases of any primary site. Since that time, more recent seminal work by Sperduto et al.^6^ resulted in the development of diagnosis-specific graded prognostic assessments utilizing a large multi-institutional cohort of nearly 4000 patients from 1985 to 2007 specific to lung, melanoma, breast, renal cell, and gastrointestinal primaries. Similar to both of the prior mentioned studies that found that extracranial metastases conferred worse prognosis among patients with lung cancer and brain metastases, we noted that patients with brain metastases of ages of 65 years and older (HR = 1.60 vs 1.46) in addition to those with synchronous lung, bone, or liver metastases (HR = 1.61 vs 1.49) had poorer OS.

Regarding potential changes in prognosis over time, a report by Nieder et al.^30^ compared a recent series of 103 patients treated from 2005 to 2009 to a cohort of 103 patients treated in 1983–1989. A higher proportion of patients in the recent cohort were noted to have received, surgery, stereotactic radiosurgery, and systemic therapies. Higher 1-year OS rates were found among the recently treated cohort (34% vs 15%; *P* = .03), but the authors noted this was likely due to a higher proportion of patients having favorable prognosis, with minimal OS improvements in patients with poorer prognoses. Our study noted significantly improved OS for patients with extracranial metastases alone versus those with synchronous brain metastases, suggesting that the general prognosis of patients with brain metastases continues to remain quite poor. However, we did find that patients diagnosed more recently had improved OS (HR = 0.98 for each year following 2010). This may be due to the changing landscape of management of brain metastases, which is evolving to include multimodality approaches of surgery, radiation therapy via either stereotactic radiosurgery or whole-brain radiation therapy, as well novel systemic therapies such as mutation-specific agents or immunotherapy with improved penetration of the blood-brain barrier.^31^

There are some limitations to our study that merit attention. First are limitations inherent to the use of the SEER database. Limitations with regard to epidemiologic studies include demographic differences (over-representation of foreign-born patients and urban inhabitants as well as non-white patients as compared to the standard US population).^17,18^ Other limitations inherent to analyses utilizing the SEER database include a lack of information on the incompleteness of patient-level data such as socioeconomic status, additional comorbidities, tumor recurrences following initial diagnosis, or intent, dose, or duration of either chemotherapy or radiation therapy, and potential for loss for information as patients move in and out of SEER geographic areas.^16–18^ Another drawback of any epidemiologic study utilizing the SEER database is that patients are logged into the database only at the time of initial diagnosis. As such, this precludes the inclusion of patients who at initial diagnosis may have early or locally advanced disease but may have developed brain metastases later on in their disease course, thus biasing our incidence value to likely be an underestimate. Other relevant information was also not available, such as the number and size of brain metastases, whether patients were symptomatic or asymptomatic at diagnosis, local control of brain metastases following treatment, and management of brain metastases following diagnosis. Also, given that screening for brain metastases is generally indicated for locally advanced lung cancers, breast cancers, and melanoma, this may bias the estimate of the relative proportion of these primary sites to be higher. Finally, as not all healthcare institutions participate in SEER, there is the concern regarding the generalizability of our findings, though SEER does comprise a large proportion (over 34%) of all patients in the United States.^16^

## Conclusions

The incidence of brain metastases from 2010 to 2015 was relatively stable at 7/100 000 patients, with 80% from lung cancers. Common primaries among pediatric patients included those of the kidney/renal pelvis and melanomas, while those in those older than 40 years of age were mostly from lung cancer. Patients with brain metastases from breast cancers had higher OS as compared to other common primary sites. Significantly poorer OS was associated with synchronous brain metastases as compared to extracranial metastases alone. Among patients with brain metastases, males, elderly patients, those with synchronous lung, bone, or liver metastases, and those treated earlier during the studied time period had poorer OS. Additional studies are warranted to further characterize the modern landscape of brain metastases in the United States to examine the incidence of patients who develop brain metastases following initial diagnosis, differential prognosis based on primary site, and an exploration of poorer OS noted among patients with synchronous brain metastases as compared to extracranial metastases alone.

## Data Availability

The data utilized for this study are provided in the SEER database (https://seer.cancer.gov/seerstat/), which is freely accessible to the public available via the National Cancer Institute SEER program, thus making the study exempt from institutional review board review.

## Supplementary Material

vdaa041_suppl_Supplementary_Material

## Notes

**Authorship Statement**. N.G.Z.: conceived idea for the project. R.S., K.C.S., H.C., E.J.L., and S.R.H.: conducted statistical analyses. R.S. and K.C.S.: drafted manuscript. R.S., K.C.S., H.C., A.V.L., E.J.L., S.R.H., J.D.P., D.M.T., P.D.B., and N.G.Z.: editing of the manuscript. All authors: final approval of the manuscript.

## References

[1] Johnson JD, Young B Demographics of brain metastasis. Neurosurg Clin N Am. 1996;7(3):337–344. 8823767

[2] Nayak L, Lee EQ, Wen PY Epidemiology of brain metastases. Curr Oncol Rep. 2012;14(1):48–54. 22012633 10.1007/s11912-011-0203-y

[3] Fox BD, Cheung VJ, Patel AJ, Suki D, Rao G Epidemiology of metastatic brain tumors. Neurosurg Clin N Am. 2011;22(1):1–6, v. 21109143 10.1016/j.nec.2010.08.007

[4] Bouffet E, Doumi N, Thiesse P, et al. Brain metastases in children with solid tumors. Cancer. 1997;79(2):403–410. 9010115 10.1002/(sici)1097-0142(19970115)79:2<403::aid-cncr25>3.0.co;2-3

[5] Graus F, Walker RW, Allen JC Brain metastases in children. J Pediatr. 1983;103(4):558–561. 6620015 10.1016/s0022-3476(83)80583-6

[6] Sperduto PW, Kased N, Roberge D, et al. Summary report on the graded prognostic assessment: an accurate and facile diagnosis-specific tool to estimate survival for patients with brain metastases. J Clin Oncol. 2012;30(4):419–425. 22203767 10.1200/JCO.2011.38.0527 PMC3269967

[7] Kocher M, Soffietti R, Abacioglu U, et al. Adjuvant whole-brain radiotherapy versus observation after radiosurgery or surgical resection of one to three cerebral metastases: results of the EORTC 22952-26001 study. J Clin Oncol. 2011;29(2):134–141. 21041710 10.1200/JCO.2010.30.1655 PMC3058272

[8] Aoyama H, Shirato H, Tago M, et al. Stereotactic radiosurgery plus whole-brain radiation therapy vs stereotactic radiosurgery alone for treatment of brain metastases: a randomized controlled trial. JAMA. 2006;295(21):2483–2491. 16757720 10.1001/jama.295.21.2483

[9] Brown PD, Jaeckle K, Ballman KV, et al. Effect of radiosurgery alone vs radiosurgery with whole brain radiation therapy on cognitive function in patients with 1 to 3 brain metastases: a randomized clinical trial. JAMA. 2016;316(4):401–409. 27458945 10.1001/jama.2016.9839 PMC5313044

[10] Crivellari D, Pagani O, Veronesi A, et al.; International Breast Cancer Study Group High incidence of central nervous system involvement in patients with metastatic or locally advanced breast cancer treated with epirubicin and docetaxel. Ann Oncol. 2001;12(3):353–356. 11332148 10.1023/a:1011132609055

[11] Sundermeyer ML, Meropol NJ, Rogatko A, Wang H, Cohen SJ Changing patterns of bone and brain metastases in patients with colorectal cancer. Clin Colorectal Cancer. 2005;5(2):108–113. 16098251 10.3816/ccc.2005.n.022

[12] Mamon HJ, Yeap BY, Jänne PA, et al. High risk of brain metastases in surgically staged IIIA non-small-cell lung cancer patients treated with surgery, chemotherapy, and radiation. J Clin Oncol. 2005;23(7):1530–1537. 15735128 10.1200/JCO.2005.04.123

[13] Gaspar L, Scott C, Rotman M, et al. Recursive partitioning analysis (RPA) of prognostic factors in three Radiation Therapy Oncology Group (RTOG) brain metastases trials. Int J Radiat Oncol Biol Phys. 1997;37(4):745–751. 9128946 10.1016/s0360-3016(96)00619-0

[14] Barnholtz-Sloan JS, Sloan AE, Davis FG, Vigneau FD, Lai P, Sawaya RE Incidence proportions of brain metastases in patients diagnosed (1973 to 2001) in the Metropolitan Detroit Cancer Surveillance System. J Clin Oncol. 2004;22(14):2865–2872. 15254054 10.1200/JCO.2004.12.149

[15] Schouten LJ, Rutten J, Huveneers HA, Twijnstra A Incidence of brain metastases in a cohort of patients with carcinoma of the breast, colon, kidney, and lung and melanoma. Cancer. 2002;94(10):2698–2705. 12173339 10.1002/cncr.10541

[16] Surveillance, Epidemiology, and End Results (SEER) Program (www.seer.cancer.gov) Research Data (1973–2012). National Cancer Institute, DCCPS, Surveillance Research Program, Surveillance Systems.

[17] Yu JB, Gross CP, Wilson LD, Smith BD NCI SEER public-use data: applications and limitations in oncology research. Oncology (Williston Park). 2009;23(3):288–295. 19418830

[18] Duggan MA, Anderson WF, Altekruse S, Penberthy L, Sherman ME The Surveillance, Epidemiology, and End Results (SEER) program and pathology: toward strengthening the critical relationship. Am J Surg Pathol. 2016;40(12):e94–e102. 27740970 10.1097/PAS.0000000000000749 PMC5106320

[19] Shuster JJ Median follow-up in clinical trials. J Clin Oncol. 1991;9(1):191–192. 1985169 10.1200/JCO.1991.9.1.191

[20] Holm S A simple sequentially rejective multiple test procedure. Scand J Stat. 1979;6(2):65–70.

[21] Bender R, Lange S Adjusting for multiple testing—when and how? J Clin Epidemiol. 2001;54(4):343–349. 11297884 10.1016/s0895-4356(00)00314-0

[22] Nieder C, Spanne O, Mehta MP, Grosu AL, Geinitz H Incidence and prognosis of patients with brain metastases at diagnosis of systemic malignancy: a population-based study. Neuro Oncol. 2017;19(11):1511–1521. 28444227 10.1093/neuonc/nox077 PMC5737512

[23] Percy AK, Elveback LR, Okazaki H, Kurland LT Neoplasms of the central nervous system. Epidemiologic considerations. Neurology. 1972;22(1):40–48. 5061838 10.1212/wnl.22.1.40

[24] Walker AE, Robins M, Weinfeld FD Epidemiology of brain tumors: the national survey of intracranial neoplasms. Neurology. 1985;35(2):219–226. 3969210 10.1212/wnl.35.2.219

[25] Counsell CE, Collie DA, Grant R Incidence of intracranial tumours in the Lothian region of Scotland, 1989-90. J Neurol Neurosurg Psychiatry. 1996;61(2):143–150. 8708681 10.1136/jnnp.61.2.143 PMC1073987

[26] Berghoff AS, Schur S, Füreder LM, et al. Descriptive statistical analysis of a real life cohort of 2419 patients with brain metastases of solid cancers. ESMO Open. 2016;1(2):e000024. 27843591 10.1136/esmoopen-2015-000024 PMC5070252

[27] Cagney DN, Martin AM, Catalano PJ, et al. Incidence and prognosis of patients with brain metastases at diagnosis of systemic malignancy: a population-based study. Neuro Oncol. 2017;19(11):1511–1521. 28444227 10.1093/neuonc/nox077 PMC5737512

[28] Kromer C, Xu J, Ostrom QT, et al Estimating the annual frequency of synchronous brain metastasis in the United States 2010–2013: a population-based study. J Neurooncol. 2017;134(1):55–64. 28567587 10.1007/s11060-017-2516-7

[29] Curless RG, Toledano SR, Ragheb J, Cleveland WW, Falcone S Hematogenous brain metastasis in children. Pediatr Neurol. 2002;26(3):219–221. 11955930 10.1016/s0887-8994(01)00363-0

[30] Nieder C, Spanne O, Mehta MP, Grosu AL, Geinitz H Presentation, patterns of care, and survival in patients with brain metastases: what has changed in the last 20 years? Cancer. 2011;117(11):2505–2512. 24048799 10.1002/cncr.25707

[31] Fecci PE, Champion CD, Hoj J, et al. The evolving modern management of brain metastasis. Clin Cancer Res. 2019;25(22):6570–6580. 31213459 10.1158/1078-0432.CCR-18-1624 PMC8258430

