# Supplementary material for: Epidemiology of synchronous brain metastases
Source: Neurooncol Adv. 2020 Apr 24;2(1):vdaa041. doi: 10.1093/noajnl/vdaa041 (PMC7182307; doi:10.1093/noajnl/vdaa041)
Supplement: vdaa041_suppl_Supplementary_Material [file vdaa041_suppl_supplementary_material.docx]

**Supplementary Tables and Figures**

Supplementary Figure 1: Flowchart of patient selection for analysis from SEER (2010-2015)

Supplementary Figure 2: Incidence of brain metastases from SEER (2010-2015) among lung and bronchus patients by non-small cell lung cancer (NSCLC) or small-cell lung cancer (SCLC)

Supplementary Figure 3: Kaplan-Meier curves examining overall survival (OS) by most common primary cancer sites

Supplementary Figure 4: Kaplan-Meier curves examining overall survival (OS) by additional primary cancer sites

Supplementary Figure 5: Kaplan-Meier curves examining overall survival (OS) for non-small cell lung cancer (NSCLC) or small-cell lung cancer (SCLC)

Supplementary Figure 6: Kaplan-Meier curves examining overall survival (OS) for breast cancer patients by hormonal receptor (HR) and HER2-neu status

Supplementary Figure 7: Log(hazard ratio) vs. time plot for overall multivariable Cox

regression model assessing the effect of synchronous brain metastases on the overall survival of

metastatic patients. Dashed lines: upper and lower 95% confidence intervals of log(hazard ratio).

Supplementary Figure 8: Log(hazard ratio) vs. time plot for site-specific multivariable Cox

regression models assessing the effect of synchronous brain metastases on the overall survival (OS) of metastatic patients by primary disease site. Dashed lines: upper and lower 95% confidence intervals of log(hazard ratio).

Supplementary Table 1: Patient characteristics of those with brain metastases at primary cancer diagnosis by age, race, sex, and cancer site

Supplementary Table 2: Summary of incidence per 100,000, as well as annual percentage change (APC) by common primary cancer sites, broken down by year of diagnosis

Supplementary Table 3: Summary of age-adjusted incidence per 100,000, as well as annual percentage change (APC) by age group, broken down by year of diagnosis

Supplementary Table 4: Summary of baseline characteristics of patients with metastatic disease either with or without synchronous brain metastases
